# Integrating acute stroke telemedicine consultations into specialists’ usual practice: a qualitative analysis comparing the experience of Australia and the United Kingdom

**DOI:** 10.1186/s12913-017-2694-1

**Published:** 2017-11-21

**Authors:** Kathleen L. Bagot, Dominique A. Cadilhac, Christopher F. Bladin, Caroline L. Watkins, Michelle Vu, Geoffrey A. Donnan, Helen M. Dewey, Hedley C. A. Emsley, D. Paul Davies, Elaine Day, Gary A. Ford, Christopher I. Price, Carl R. May, Alison S. R. McLoughlin, Josephine M. E. Gibson, Catherine E. Lightbody, on behalf of the VST and ASTUTE investigators

**Affiliations:** 1 Stroke Division, The Florey Institute of Neuroscience and Mental Health, University of Melbourne, Melbourne, VIC Australia; 2 University of Central Lancashire, Preston, UK; 3 Department of Medicine, School of Clinical Sciences at Monash Health, Monash University, Melbourne, Australia; 4 Australian Catholic University, Sydney, Australia; 5 Eastern Health Clinical School, Monash University, Melbourne, Australia; 6 Lancashire Teaching Hospitals NHS Foundation Trust, Preston, UK; 7 North Cumbria University Hospitals NHS Trust, Carlisle, UK; 8 Lancashire and South Cumbria Strategic Clinical Network, Greater Manchester, UK; 9 Oxford University Hospitals NHS Foundation Trust and Division of Medical Sciences, University of Oxford, Oxford, UK; 10 Northumbria Healthcare NHS Foundation Trust, Newcastle, UK; 11 Newcastle University, Newcastle, UK; 12 University of Southampton, Southampton, UK

**Keywords:** Telemedicine, Normalisation process theory, Implementation, Barriers, Facilitators, Acute, Consultation

## Abstract

**Background:**

Stroke telemedicine can reduce healthcare inequities by increasing access to specialists. Successful telemedicine networks require specialists adapting clinical practice to provide remote consultations. Variation in experiences of specialists between different countries is unknown. To support future implementation, we compared perceptions of Australian and United Kingdom specialists providing remote acute stroke consultations.

**Methods:**

Specialist participants were identified using purposive sampling from two new services: Australia’s Victorian Stroke Telemedicine Program (*n* = 6; 2010–13) and the United Kingdom’s Cumbria and Lancashire telestroke network (*n* = 5; 2010–2012). Semi-structured interviews were conducted pre- and post-implementation, recorded and transcribed verbatim. Deductive thematic and content analysis (NVivo) was undertaken by two independent coders using Normalisation Process Theory to explore integration of telemedicine into practice. Agreement between coders was M = 91%, SD = 9 and weighted average κ = 0.70.

**Results:**

Cross-cultural similarities and differences were found. In both countries, specialists described old and new consulting practices, the purpose and value of telemedicine systems, and concerns regarding confidence in the assessment and diagnostic skills of unknown colleagues requesting telemedicine support. Australian specialists discussed how remote consultations impacted on usual roles and suggested future improvements, while United Kingdom specialists discussed system governance, policy and procedures.

**Conclusion:**

Australian and United Kingdom specialists reported telemedicine required changes in work practice and development of new skills. Both groups described potential for improvements in stroke telemedicine systems with Australian specialists more focused on role change and the United Kingdom on system governance issues. Future research should examine if cross-cultural variation reflects different models of care and extends to other networks.

## Background

Telemedicine can increase access to hyper-acute stroke care (i.e., emergency treatment available only within 4.5 h of symptom onset) in places where there are a limited number of stroke specialists, including rural areas. Stroke specialist assessment (e.g. neurologists or stroke physicians) improves accessibility to, and rapid delivery of, thrombolysis [1, 2]. In turn, patient outcomes are improved, including the increased likelihood of being discharged to home, with independent ambulation and reduced disability days [1, 2]. Compared to bedside specialists, evaluation results show telemedicine for acute stroke is safe (i.e. no significant difference in post-treatment intracerebral haemorrhage or mortality rates), reliable and valid (i.e. similar patient assessment, including stroke severity) and cost effective (i.e., from hospital and social perspectives) [1, 3]. Exploration of barriers and enablers to implementation have generally focused on organizational factors (e.g. licensing, credentialing, reimbursement and malpractice liability) [4, 5], and which models to use [6]. Whilst these studies provide insights into organisational practices and policies, there are few well-designed process evaluations to provide insights into how telemedicine is embedded and integrated into practice [7].

Successful implementation of telemedicine services often requires clinicians and specialists adapting practices to incorporate acute clinical consultations outside of their health network. Although stroke telemedicine is practised internationally, very little is known about clinicians’ experiences in the delivery of healthcare via audio-visual, technology-based platforms. Such disruptive innovation [8] clearly changes specialists’ usual practice. Preliminary research from Australia has indicated that specialists involved in acute stroke telemedicine consultations have both negative (e.g. impacts personal life) and positive (e.g. improves healthcare equity) experiences [9]. These experiences are likely to affect specialists’ willingness to continue involvement in telemedicine networks. Further, the working relationships between healthcare professionals within telemedicine networks are major factors of a successful American network [4] and new Australian network [10]. Working with local clinicians who vary in acute stroke expertise can be difficult for remote specialists [7, 9].

The current literature is limited in its presentation of factors for new services to consider, reducing the likelihood of future network implementation success. Significant yet unidentified differences between contexts may exist and the question remains whether implementation factors in one setting are relevant in another setting. In particular, the extent to which specialists’ experiences with telemedicine vary between telestroke networks remains unknown. This study compared the perceptions of Australian and the United Kingdom (UK) specialists’ experiences in providing remote telemedicine consultations for acute stroke patients.

## Method

Method and results are reported using the Consolidated Criteria for Reporting Qualitative studies (COREQ) [11]. Ethics approval was received from the Human Research Ethics Committees of the Australian regional hospital, the National Research Ethics Service (UK) and the University of Central Lancashire’s, Buildings, Sport and Health Ethics Committee.

### Design and setting

A pre-post qualitative study was conducted with telestroke networks from Australia (Victorian Stroke Telemedicine [VST] Program) [12] and the UK (Cumbria and Lancashire telestroke network) [7]. Both networks had mobile telemedicine carts in participating hospitals for use at the patient’s bedside, allowing a two-way audio-video connection to specialists, and for brain images to be viewed remotely. Training was provided in how to access images and use the telemedicine equipment Additional training in CT scan interpretation was undertaken in the UK. Training strategy and programme for the UK can be accessed at http://www.astute-telestroke.org.uk/section4.htm

The VST Program commenced with a single pilot hospital in 2011 [13] covering a population of 308,000 and is now active in a further 15 hospitals across five regions in Victoria [14], a south-eastern state of Australia. A virtual hub of 12 metropolitan-based stroke specialists (all consultant neurologists) provide a 24/7/365 service. A consult payment schedule comprising a daily on-call rate and fee-for-service is used and the on-call role is in addition to their usual duties. Specialists are on-call for 24 h shifts from 8 am; one specialist covers the weekend. Patients presenting to participating regional emergency departments within 4.5 h of suspected stroke onset are eligible. Between July 2011–September 2016, there were 1001 initial telemedicine consults with a further 141 follow-up consults conducted by VST neurologists. Of these, 235 were recommended for thrombolysis and 46 for endovascular clot retrieval.

The UK contribution to this study was part of a multi-phase project: the Acute Stroke Telemedicine: Utility, Training and Evaluation (ASTUTE) [7, 15, 16]. This project ran alongside the development and implementation of the Lancashire and Cumbria Telestroke Network, and produced a standardised telemedicine toolkit (http://www.astute-telestroke.org.uk). The Lancashire and Cumbria Telestroke Network was launched in August 2011, comprising eight hospitals, covering a population of nearly 2.2 million. Fifteen stroke specialists (i.e. stroke geriatricians, neurologists, medical consultants), participate in an on-call rota covering nights, weekends and public holidays. The stroke specialists are on-call from 5 pm until 8 am weekdays and for 24 h shifts at weekends and public holidays. The on-call is incorporated into the specialist’s job plan (i.e. forms part of their usual duties) negotiated at each Trust. Between July 2011–September 2016, there were 1503 telestroke assessments; 672 received thrombolysis.

### Participants and procedure

Participants were the specialists providing the telestroke consultations. Participants were identified using maximum variation sampling to try and reflect exposure in terms of sex, clinical experience and geographical location of rostered specialists for both networks. Specialists involved in the development and implementation of each network were approached face-to-face, through telephone or email by network staff and invited to participate in an interview; all agreed.

Semi-structured interviews (using schedules outlining questions and probes used are available from first author) were conducted face-to-face or via telephone with three specialists from each network both pre- and 12 months post-implementation. The pre-implementation interview schedules covered participants’ prior experience with telemedicine, obstacles for implementing the network, and how it might impact on performing their current role. The post-implementation interview schedules included their level of comfort in making decisions via telemedicine consultations, service improvements that could be made, and identifying future applications of telemedicine. The schedule content was reviewed by a stroke specialist ensuring relevance and face validity. Experienced researchers (all female) with qualitative interview expertise, who may or may not have been known to the participant, performed the interviews with no other project personnel present. Interviews were audio-recorded with participant’s consent, transcribed verbatim and de-identified for analysis. Field-notes were taken. No repeat interviews were carried out, and transcripts were not returned to participants for comment, except two UK pre-implementation transcripts (no edits required).

### Analysis plan

Deductive thematic and content analyses were undertaken by two independent coders (Australia KB; UK CL) within NVivo10 [17]. The Normalisation Process Theory (NPT) [18] coding framework was used. NPT was designed to explore the integration of interventions into practice [19] including the assimilation of complex e-health initiatives [20]. The four NPT components (Coherence, Cognitive Participation, Collection Action, Reflexive Monitoring) have four sub-components; detailed in Results. A coding protocol was developed, trialled and refined using an additional transcript from each network. Discussions were ongoing throughout coding ensuring consistent application.

The mean number of comments (i.e. total number of comments divided by number of participants) assigned to each NPT sub-component was calculated by sub-component, pre- and post-implementation. These data are presented in radar plots which depict the distribution of comments across NPT components, allowing comparisons between networks (Australia blue, UK red), and also pre- (solid line) and post- (dotted line) implementation. The more comments made within a sub-component, the further out the point is extended on the plot axis.

## Results

The final sample for analysis consisted of six interviews (conducted 2010–2013) from five Australian specialists and five interviews (conducted 2010–2012) from five UK specialists; a total of *N* = 10 specialists. Technical issues meant one pre-implementation UK interview was unavailable; the field-notes for this interview suggested issues raised were similar to other UK interviews.

**Fig. 1:**
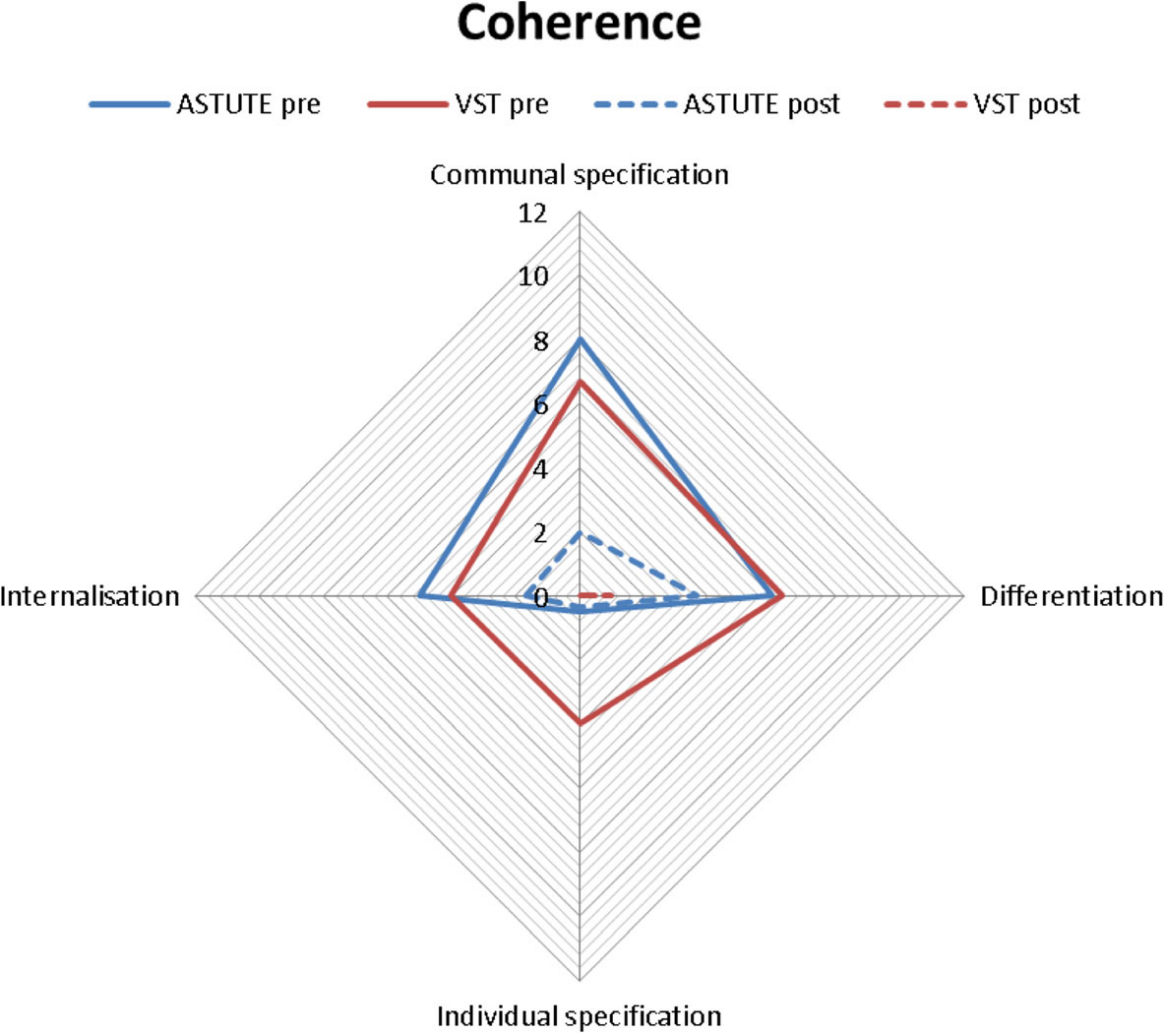
Coherence.  UK pre-implementation;  Australia pre-implementation;  UK post-implementation;  Australia post-implementation

The first component, **Coherence,** is the process that people go through when attempting to understand a new set of practices; that is, sense-making work (Fig. 1). Pre-implementation, Australian and UK specialists reported similar levels for three of four sub-components of Coherence. Australian specialists more frequently commented on understanding what telestroke required of them (individual specification), with UK specialists barely mentioning this. The comments mostly focused on examining the purpose and function of the telestroke system (communal specification), the benefits and importance of telestroke (internalisation), and perceived differences between old and new systems of work (differentiation).

Differentiation example: *“… it’s really difficult not touching people because I would normally go to a patient and say ‘hello I’m [name]’… of course I can say that on the tele-conferencing but I can’t take hold of their hand as I normally would or kind of use the appropriate body language so it’s, it is a bit tricky, …”* (UK specialist 1, pre-implementation)

**Fig. 2:**
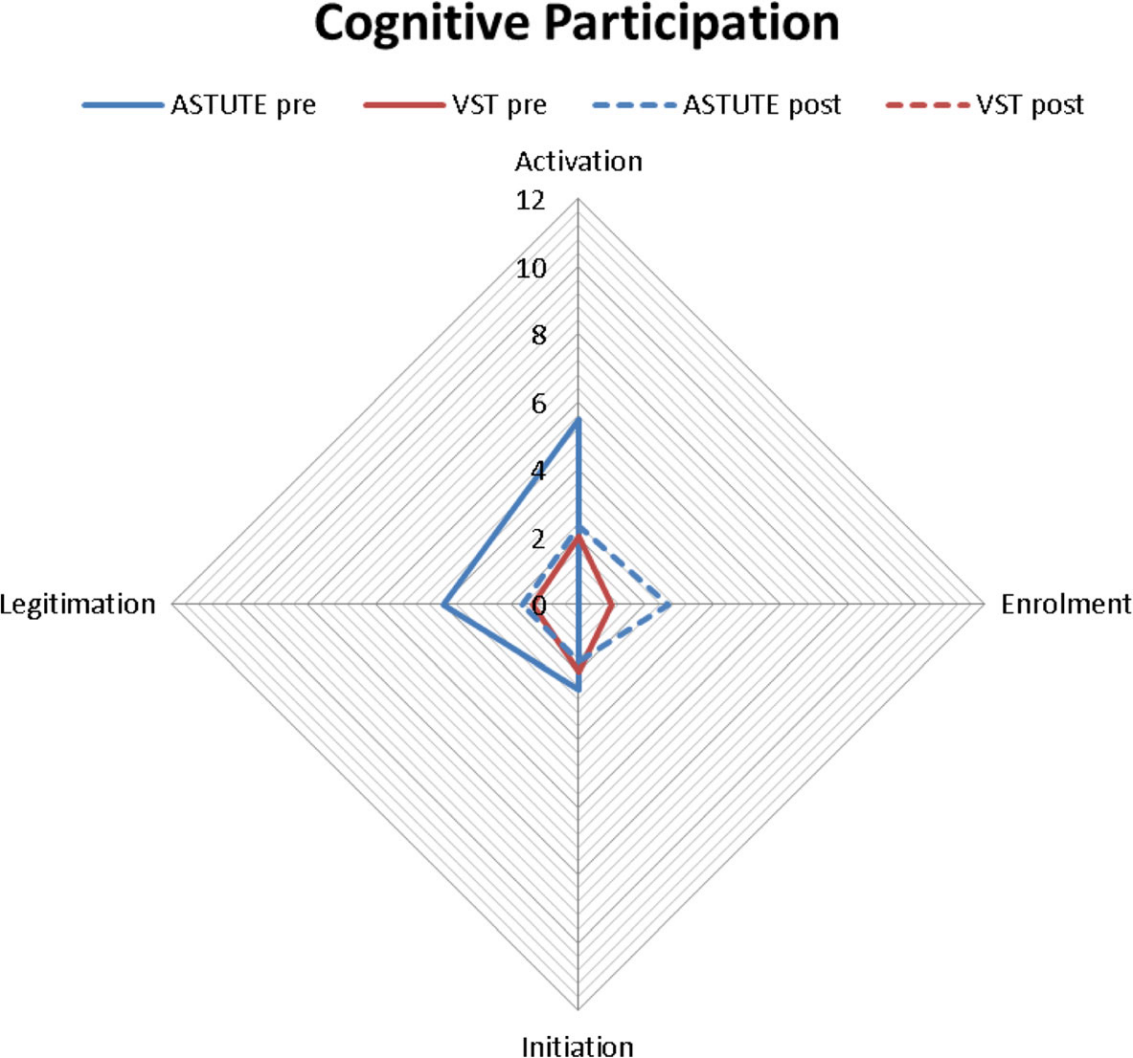
Cognitive Participation.  UK pre-implementation;  Australia pre-implementation;  UK post-implementation;  Australia post-implementation

The second component, **Cognitive Participation,** is ‘the relational work that people do to build and sustain a new practice’ and received the fewest comments (Fig. 2). The UK specialists made the majority of comments pre- and post-implementation, with only one or two comments by Australian specialists pre- and zero comments post-implementation. Prior to implementation, UK specialists most frequently commented on developing relevant policies and procedures (activation), and system governance considerations (e.g. reimbursement, legal requirements) for introducing telemedicine (legitimation). There were comments from both groups about factors driving telestroke implementation forward (initiation). There were fewer comments on these sub-components post-implementation; UK specialists commenting that stroke telemedicine should be part of their work (enrolment).

Activation example: *“Is there any reason why we can’t do a big video conferencing from the people who are going to be involved with VST [Australian telemedicine network]? … Then everyone knows the faces beforehand of the consultants who will be involved, the A and E [Accident and Emergency Department] consultants who will be involved, any registrars who’s (sic) available and the consultants here who will be involved.”* (Australian specialist 1, pre-implementation)

**Fig. 3:**
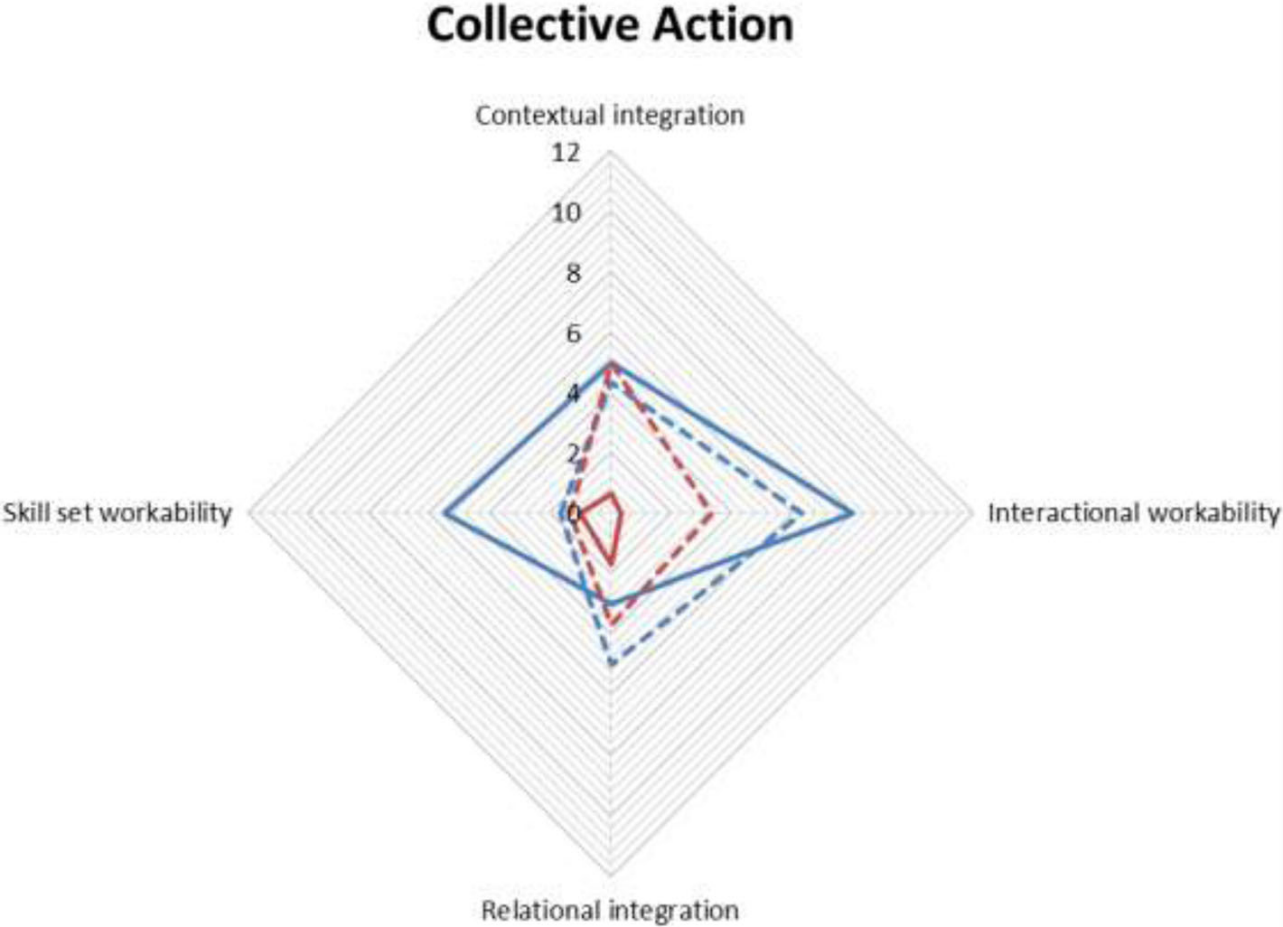
Collective Action.  UK pre-implementation;  Australia pre-implementation;  UK post-implementation;  Australia post-implementation

The third component, **Collective Action,** is the operational work that people do to enact a new practice. UK specialists made more comments than Australian specialists (Fig. 3). Pre-implementation, UK specialists focussed on skills and training (skill set workability) but after experiencing telemedicine, they were concerned with confidence in themselves and others’ abilities (relational integration). There were similar numbers of comments across pre- and post-implementation about staff and patients’ ability to perform the tasks required for telemedicine (interactional workability). UK and Australian specialists made similar comments regarding organisational support and resourcing, such as leadership and training (contextual integration).

Interactional workability example: *“…I do [a] very busy acute medicine job, and if I am thrombolysing somebody at half past two in the night then I’m expected to come back at eight o’ clock to do my ward round that can become a little bit hard, and also unsafe for my patients if I’m half asleep so I’ve highlighted this to my health management [line manager] that I will need to have a morning off once I’ve been doing my on-calls …”* (UK specialist 2, post-implementation)

**Fig. 4:**
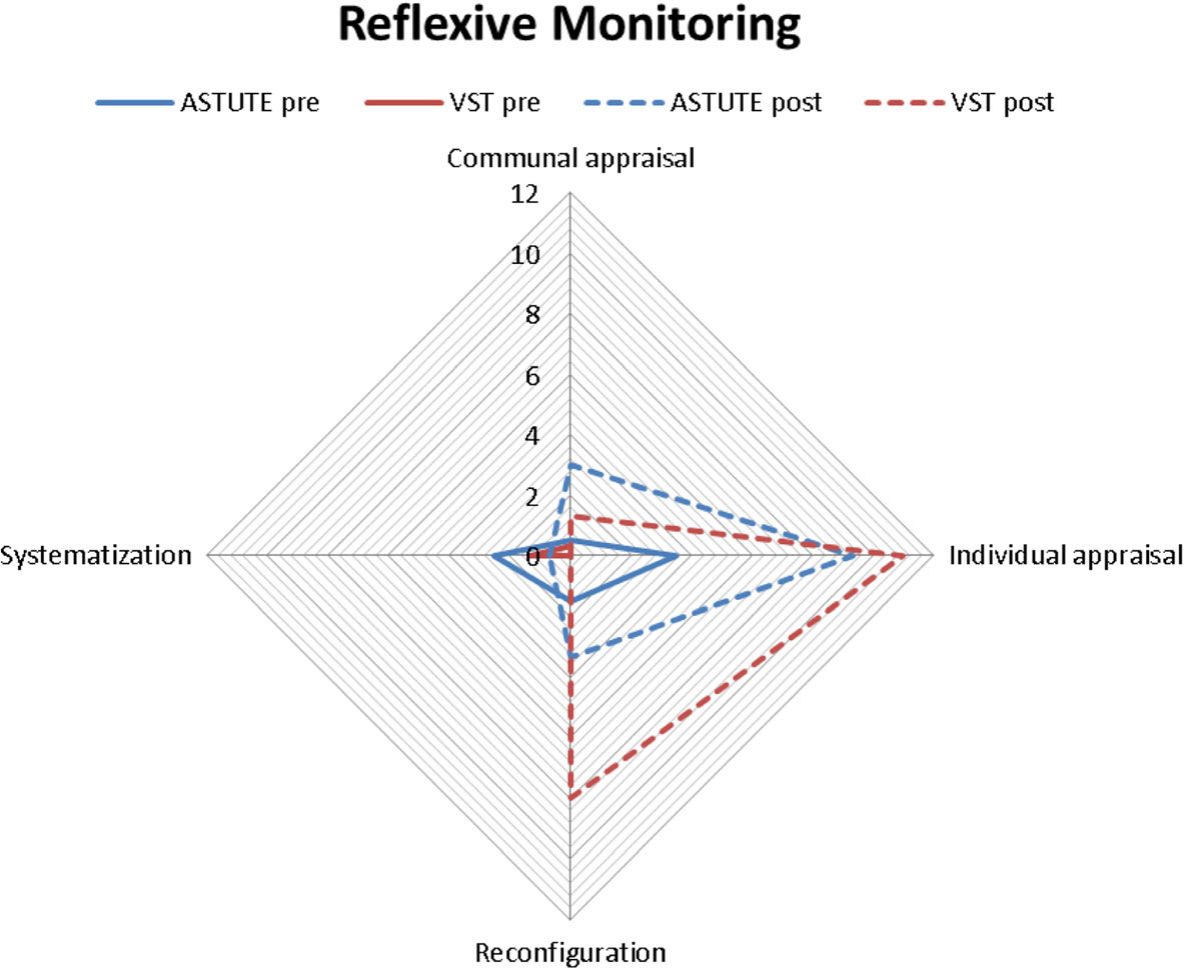
Reflexive Monitoring.  UK pre-implementation;  Australia pre-implementation;  UK post-implementation;  Australia post-implementation

The final component, **Reflexive Monitoring**, is the appraisal work undertaken to understand how new practices affect them and others (Fig. 4). Pre-implementation, there were few comments across the four sub-components, except for UK specialists’ comments evaluating telestroke (Individual appraisal). Post-implementation, both UK and Australian specialists focused on their own performance, with few comments about information collection on telemedicine system’s impact (systematisation). One UK specialist reported specific outcome measures being collected.

While both groups spoke about changes in work practices due to experience and evaluation of telestroke (individual appraisal), Australian specialists spoke more about this. Comments were made about criteria to evaluate the work of telemedicine (communal appraisal). Post-implementation, both groups commented on aspects of modification (reconfiguration), mostly to the broader telemedicine system.

Reconfiguration example *“…at the moment, we’re on-call for a day because you can slot it in with other things, but if you were getting frequent calls with the expectation that you know once an hour you’d be on the phone, then I think having a sort of half day of roster would be better from my point of view.”* (Australian specialist 2, post-implementation)

Inter-rater reliability analyses (i.e. proportion of text that raters agreed to be allocated or not to sub-components) revealed excellent agreement (Mean % agreement across components = 91%, SD = 9% with weighted average κ = 0.70. Data saturation (i.e. additional interviews yielding no new information) could not be used in considering if there was sufficient data as there was a finite number of specialist telemedicine consultants. However, as transcript content was able to be coded to all 16 NPT framework sub-components (illustrating breadth of interview content) and differences and similarities between the two networks were clearly identified, these interviews were considered sufficient.

## Discussion

Specialist’s perceptions from two countries involved in implementing new telestroke networks were compared. Similarities and differences were experienced between the two networks’ specialists suggesting that contextual factors are important for successful network implementation.

Consistent with NPT, before the telestroke networks were operational, specialists from both countries focused on understanding new systems and impact. These comments reduced post-implementation, suggesting that experience with telemedicine consultations over 12 months had allowed specialists from both networks to incorporate telemedicine into their roles (i.e., normalisation). Involving key stakeholders early in the planning process was suggested to ensure successful implementation [6]; this provides time for stakeholders to understand and prepare for changes to individual roles and organisational settings. Losing cues from face-to-face consultations and learning to trust remote staff’s skills were influential [21] and telemedicine consulting should be in the curriculum [22].

There were differences between the groups; Australian specialists commented on what telemedicine consulting required of them as individuals, while UK specialists commented on the governance procedures, clinical pathways and required resources across participating organisations. Planning, governance procedures and physician reimbursement for telemedicine have been highlighted previously as critical factors [4, 23]. These differences in the current study likely reflect how telemedicine was introduced: for UK specialists, telemedicine was incorporated into their job plan, whereas for Australian specialists, telemedicine consultations were in addition to their usual role and working with a single site. The UK network involved multiple sites, across which specialists were required to consider policies and procedures (virtual rotating hub and spoke model), heightening governance issues.

Being on-call had impacts on social plans and sleep disruption. Social impact came mostly from Australian specialists who had access to laptops and mobile internet devices. While technology facilitates workplace flexibility, work infringing into personal life has previously been identified as a barrier to implementing telestroke [5]. Disrupted sleep patterns [24], and being on-call, without being contacted, can have negative repercussions [25]. Additional specialists participating could reduce on-call frequency. A follow-the-sun model [26] where specialists in other time-zones can provide remote consultations (e.g. Australia supports UK) could limit out-of-hours disruption and support specialists’ retention.

The level of support varied within each network, with both Australian and UK specialists indicating high and low support from their home hospitals. New telemedicine networks should pay particular attention to addressing integration and adaptation factors for specialists. Although both networks had extensive evaluation programs, specialists didn’t mention evaluation or data collection. To report outcomes to relevant stakeholders, data collection is critical. Although there are quality and outcome indicator recommendations (AHA/ASA statement), an internationally agreed minimum data set for telemedicine with definitions shared should be established. This would support meaningful systemic data collection with minimum resources.

The current study’s strengths include the pre-post design, comparison of networks in two countries and the use of an established theoretical framework. Although only the UK interview schedule drew on the NPT framework, comments from Australian specialists did cover all components of NPT. One limitation of the NPT framework was that it does not specifically capture if participants had any prior experience with a similar and how that experience may influence the implementation process. Our method of coding included both quantitative (i.e. radar plots) and qualitative (i.e. narrative) presentations of the data. While both networks targeted improving access-to-care [27], the UK had a virtual rotating hub and spoke model and was operational during out of hours and weekends only, whilst Australia had a central hub and was operational 24/7. These models impact specialists’ involvement and subsequent consultation experience. This study is based on a small number of specialists focusing on early experiences of establishing a telestroke network. Future work is required to explore specialist experiences one established.

## Conclusion

Australian and UK specialists reported telemedicine required changes in work practice and development of new skills. Both groups described potential for improvements in stroke telemedicine systems with Australian specialists more focused on role change and the United Kingdom on system governance issues. The variation identified may reflect different Australia and UK models of care requiring further exploration. Future research might investigate the transferability of UK and Australian experiences to broader European, Asian and American networks.

## Abbreviations

ASTUTE: Acute Stroke Telemedicine: Utility, Training and Evaluation
NPT: Normalisation Process Theory
UK: United Kingdom
VST: Victorian Stroke Telemedicine
